# Analysis of Genetic Alteration Signatures and Prognostic Values of m6A Regulatory Genes in Head and Neck Squamous Cell Carcinoma

**DOI:** 10.3389/fonc.2020.00718

**Published:** 2020-05-29

**Authors:** Xuanchen Zhou, Jie Han, Xiaoyue Zhen, Yiqing Liu, Zhaoyang Cui, Zhiyong Yue, Ling Ding, Shuai Xu

**Affiliations:** ^1^Department of Otorhinolaryngology Head and Neck Surgery, Shandong Provincial Hospital Affiliated to Shandong First Medical University, Jinan, China; ^2^Department of Otorhinolaryngology Head and Neck Surgery, Shandong Provincial Hospital Affiliated to Shandong University, Jinan, China; ^3^Minimally Invasive Urology Center, Shandong Provincial Hospital Affiliated to Shandong First Medical University, Jinan, China

**Keywords:** N6-Methyladenosine, head and neck squamous cell carcinoma, ALKBH5, YTHDC2, prognosis, biomarkers

## Abstract

Genetic alteration involving N6-methyladenosine (m6A) regulatory genes is a frequent characteristic of multiple tumors. Nevertheless, little is known regarding their genetic alteration signatures and prognostic values in head and neck squamous cell carcinoma (HNSCC). In this study, RNA sequence profiles and copy number variation (CNV) data of 506 HNSCC patients were downloaded from The Cancer Genome Atlas (TCGA) database. Correlation analysis involving alteration of m6A regulatory genes, clinicopathological characteristics, and patient survival was performed using R language. The results suggest that alteration of m6A regulatory genes was correlated with clinical staging. Patients with high expression of *ALKBH5, FTO, METTL14, WTAP, YTHDC1, YTHDF1*, and *YTHDF2* had poor overall survival (OS) than those with low expression. Univariate and multivariate Cox regression analyses showed that *ALKBH5* and *YTHDC2* were independent risk factors for OS. However, patients with high YTHDC2 expression had better OS. Moreover, according to machine learning results, *YTHDC2* was found to be the most important gene among the 10 m6A regulators. Additionally, high expression of *YTHDC2* was correlated with activation of apoptosis and ubiquitin-mediated proteolysis. Here, we identified alterations to m6A regulatory genes in HNSCC for the first time and found that seven m6A regulators were associated with poor prognosis, especially *ALKBH5*, whereas *YTHDC2* was associated with better prognosis. These m6A-related regulators could act as novel prognostic biomarkers for HNSCC. Our findings provide clues for understanding RNA epigenetic modifications in HNSCC.

## Introduction

Head and neck squamous cell carcinoma (HNSCC) is a common clinically malignant tumor that mainly occurs on the mucous surface of the upper respiratory digestive tract, such as the nasal cavity, paranasal sinuses, nasopharynx, hypopharynx, larynx, trachea, oral cavity, and oropharynx. According to statistics, there are > 550,000 new cases of squamous cell carcinoma of the head and

neck worldwide every year (1, 2). According to “Cancer statistics in China, 2015,” the incidence of new lip, oral, and oropharyngeal cancer in 2015 was 48.1/100,000, and the mortality rate was 22.1/100,000. The incidence of new nasopharyngeal carcinoma (NPC) was 60.6/100,000, and the mortality rate was 34.1/100,000; the incidence of new laryngeal cancer was 26.4/100,000, and the mortality rate was 14.5/100,000 (3). The latest statistics show that, in the United States, HNSCC accounts for 3% of all cancer patients with 60,000 new cases per year and 12,000 deaths per year (4). The five-year survival rate of patients with HNSCC is approximately 40–50%.

At present, tobacco use and alcohol consumption are still the most important risk factors for development of HNSCC (5). There is relatively little information concerning the molecular mechanisms underlying HNSCC progression. However, in order to improve outcomes in HNSCC cases, identifying molecular genetic events during tumor progression is crucial to understanding mechanisms underlying malignancy.

The important role of RNA in biological systems is not only through the flow of genetic information from DNA to protein, but also through the regulation of various biological processes (6). The multiple functions of RNA are accompanied by more than 100 chemical modifications although the functions of most of these RNA modifications remain unclear (6). Among these modifications, adenosine N6 methylation (m6A) is considered to be the most common and conservative internal transcriptional modification in eukaryotic mRNAs (7). RNA m6A is thought to affect RNA transcription, processing, translation, and metabolism (7). The deposition of m6A is encoded by a methyltransferase complex, which involves three homologous factors, namely “writer,” “eraser,” and “reader” (7).

The “writers” (*METTL3, METTL14, WTAP*, and *RBM15/15B*) can catalyze the formation of m6A; “erasers” (*FTO* and *ALKBH5*) can selectively remove the methyl code from target mRNAs; “readers” (*YTHDF1, YTHDF2, YTHDF3, YTHDC1*, and *YTHDC2*) can decode methylation of m6A and produce a functional signal (7). m6A RNA modification is a dynamic and reversible process, which involves several biological functions in mammals, such as RNA transcription, processing events, splicing, RNA stability, and translation (8). To date, m6A has been reported to be associated with a variety of disorders, such as infectious diseases, nervous system development, obesity (9), infertility, inflammation, and cancer (10). Emerging evidence shows that m6A modification is related to tumor proliferation, differentiation, occurrence, invasion, and metastasis and plays the role of oncogene or antioncogenes in malignant tumors (7). In acute myeloid leukemia (AML), mimicking FTO depletion, an FTO inhibitor FB23-2 significantly inhibited the proliferation of human AML cell lines and primary cells *in vitro* and promoted cell differentiation/apoptosis (11). HNRNPC has been reported to control the invasiveness of glioblastoma (GBM) cells by regulating PDCD4 (12). In lung cancer, FTO enhances the expression of MZF1 by reducing the mA level and mRNA stability of MZF1 mRNA transcription, then leading to carcinogenic function (13). The deletion of m6A methyltransferase METTL3 leads to selective splicing and gene expression alteration of >20 genes involved in the TP53 signaling pathway in hepatocellular carcinoma (HCC) (14). In breast cancer (BRC), by inhibiting let-7g to upregulate METTL3, HBXIP forms a positive feedback loop of HBXIP/let-7g/METTL3/HBXIP, leading to accelerated proliferation of breast cancer cells (15). Although m6A has been found to be associated with tumorigenesis in various types of cancer, little is known regarding the relationship between m6A regulators and HNSCC. Therefore, we performed a retrospective analysis based on The Cancer Genome Atlas (TGCA) database to analyze genetic alterations involving m6A-related genes, their relationship with clinicopathological characteristics of HNSCC, and the prognostic value.

## Materials and Methods

All TCGA clinical data, copy number variations (CNVs), mutations, and mRNA expression data were retrieved using the UCSC Xena program, which is available to the public under certain guidelines. Therefore, written informed consent was obtained.

### SNP and CNV Analysis of 506 HNSCC Samples

All of the TCGA RNA-sequencing data, single nucleotide polymorphisms (SNPs) and CNV data, clinical phenotypes, and survival data of 506 samples from patients with HNSCC were preliminarily integrated and standardized and could then be directly used for subsequent analysis. SNP analysis was based on VarScan2 variant aggregation and masking data in TCGA, in particular for mutation analysis of 10 m6A regulatory genes. These 10 m6A genes were *METTL3, METTL14, WTAP, FTO, ALKBH5, YTHDF1, YTHDF2, YTHDF3, YTHDC1*, and *YTHDC2*. SNP mutation analysis and results of visualization in this study were performed using the R maftools package (16).

CNV analysis of the 10 m6A regulatory genes was performed using GISTIC 2.0 (17) software with default parameters, and the results were visualized using the R programing language.

### Relationship Involving CNVs, m6A Gene Expression, and Clinical Phenotypes

To investigate the relationship between CNVs and gene expression, we used the R package “ggpubr” to visualize the expression levels of the 10 m6A regulatory genes with or without CNVs and performed Kruskal–Wallis *H* tests between different CNV event groups. Here, expression results are represented as box plots.

To study relationships involving CNVs and clinical phenotypes, we performed analysis based on age, gender, clinical stage, tumor node metastasis (TNM) stage, and sample CNV information. Chi-square testing was performed after grouping the clinical phenotype and CNV information.

### Survival Analysis

We performed survival analysis based on regulatory gene expression levels of 10 m6A loci (log2 normalized) and overall survival time (OS) to determine the prognostic value of these 10 genes. Before survival analysis, we removed the gene expression level from the normal control sample from the original data. Samples with survival status of zero (survival) and survival time of <30 days were considered as follow-up failures in this study, and the remaining HNSCC samples were included in the subsequent survival analysis. The optimal cutoff point for gene expression was obtained based on the R package “survminer” (18). After high–low expression ranking of the samples using the optimal cutoff point, survival analysis was performed using the R package “survival” (19), and survival analysis results were visualized by the “ggsurvplot” method.

### Construction of an m6A Regulatory Gene Network

We used the geneMANIA (20) database as the main analytical tool to explore the interaction between 10 m6A regulatory genes and other genes using default parameters. Ten m6A regulatory genes were used as analytical background genes, and the functions of the genes enriched in the visualization network results were further analyzed to study the potential main cellular roles of the 10 regulatory genes.

### Machine Learning and Gene Set Enrichment Analysis (GSEA)

Based on the R package “caret,” we constructed two different machine learning models to investigate features of importance involving 10 m6A regulatory genes. Random forest and neural network models were used to rank the 10 genes by their expression levels in two disease states (tumor and normal). Results were also visualized using R language, and the most important feature of the two models was selected as the key gene. We then used GSEA software (version 4.0.1) to perform GSEA analysis of key genes and other mRNAs. The Kyoto Encyclopedia of Genes and Genomes (KEGG).v7.0 was used as a gene sets database, and the Pearson method was selected as a metric for ranking genes while other parameters were used at default settings. Among the final enrichment results, *p* < 0.05 was used as the statistically significant cutoff to analyze optimal pathway enrichment results.

### Immunohistochemistry (IHC)

According to the Abcam manufacturer's instructions, the *YTHDC2* and *ALKBH5* genes were analyzed by IHC using formalin-fixed, paraffin-embedded tissue blocks. Briefly, paraffin sections were dewaxed, and endogenous peroxidase activity was blocked with 0.3% hydrogen peroxide for 10 min at 37°C. Following antigen retrieval, the tissue sections were incubated with primary antibody *YTHDC2* (1:500; EPR21820-49, AB220160; Abcam, Inc., Cambridge, MA, USA) or *ALKBH5* (1:2000; EPR18958, ab195377, Abcam, Inc.) at 4°C overnight and then with a secondary antibody at room temperature (25°C) overnight. The sections were counterstained with hematoxylin.

## Results

### SNP Mutation and CNV Results of m6A Regulatory Genes in HNSCC Samples

**Figure 1:**
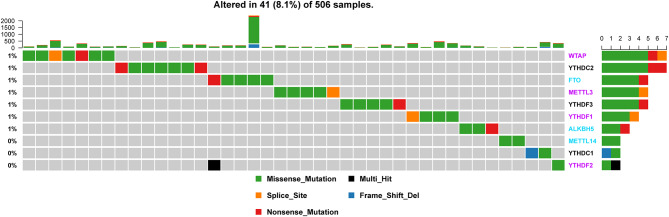
Alteration frequency of m6A regulatory genes in the HNSCC sample based on the TCGA database.

**Figure 2:**
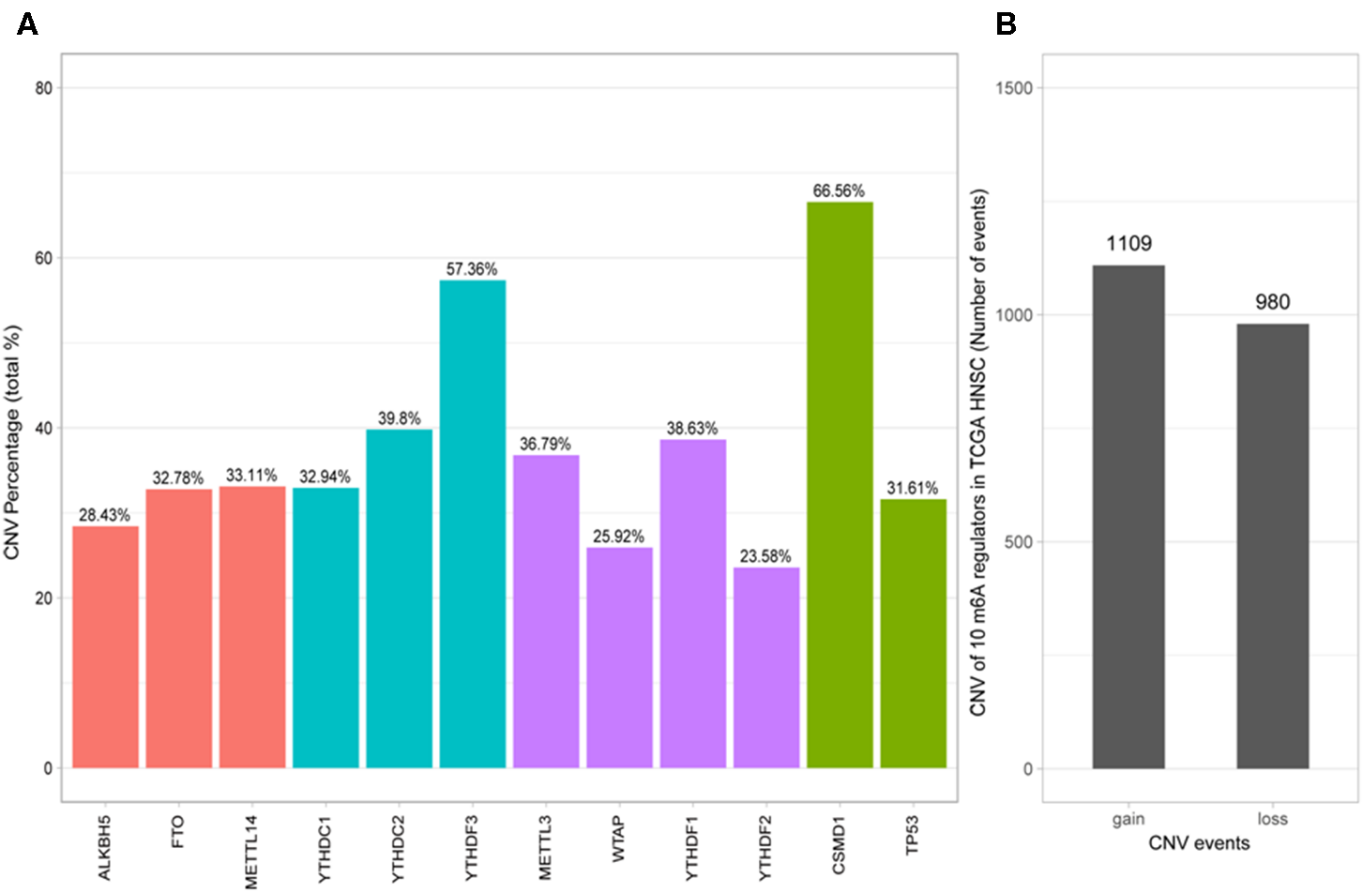
Analysis of 10 m6A regulatory gene mutation data in the TCGA HNSCC sample. **(A)** Percentage of HNSCC samples with CNVs of the m6A regulatory genes from TCGA. **(B)** CNV events of gain or loss of m6A regulators in HNSCCC samples.

Of the 506 patients with sequencing data used for SNP mutation analysis, only 41 (8.1%) of the samples had mutation events in any of the 10 m6A regulatory genes as shown in Figure 1. For HNSCC samples, the mutation frequency involving the 10 m6A regulatory genes was not high, and each gene was mutated only in several samples (<8), where most of the mutation events were missense mutations. However, it was found that the 10 m6A regulatory genes had different levels of CNV events though analysis using GISTIC, ranging from 23.58 to 57.36%. “Reader” gene *YTHDF3* (57.36%) harbored the most CNV events among the 10 m6A genes (Figure 2A). CSMD1 (66.56%) and TP53 (31.61%) were non-m6A genes found to be mutated while CSMD1 was the most CNV events and TP53 was the most SNP events in all HNSCC samples. They were analyzed together with 10 m6A regulatory genes as references.

**Figure 3:**
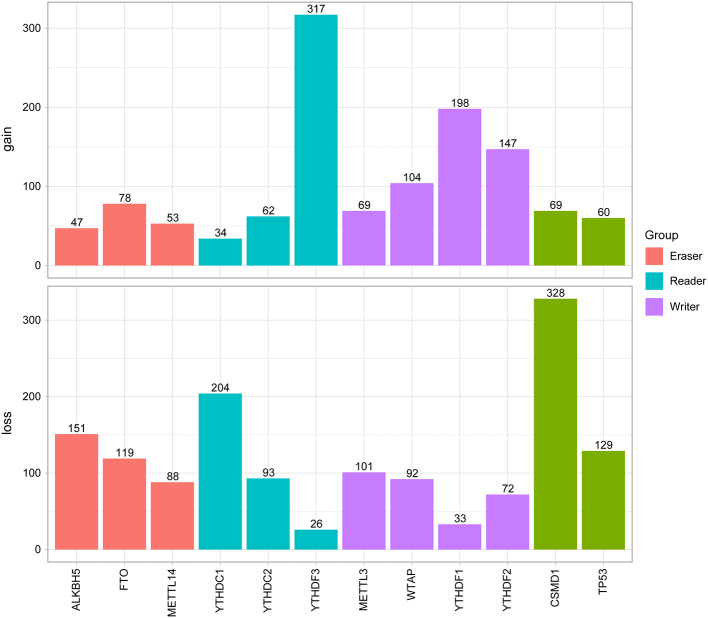
Results of CNV analysis of 10 m6A regulatory genes in the HNSCC cohort.

Next, the CNV patterns of the 10 m6A genes in the HNSCC samples were analyzed. It was found that the number of the CNV gain event was larger than that of loss events (1109 > 980) in the 10 m6A regulatory genes (Figure 2B). Unlike AML (21) and clear cell renal cell carcinoma (ccRCC) (22), among these 10 m6A regulatory genes, *YTHDF3* exhibited the highest number (317) of CNV gain events. *YTHDC1* had the most CNV loss events with 204 cases (Figure 3). Both *YTHDF3* and *YTHDC1* are m6A regulatory “reader” genes.

Additionally, SNP analysis revealed that the most frequent variant classification of m6A regulatory gene mutation was missense mutation, and the largest number of variant types was SNP (Figure S1). For SNPs, the most common SNV type was C > T, and the median number of variants per sample was 78. The most obvious mutations involved *TP53* as mentioned before (66%) and *TTN* (35%).

### Association Involving CNV Alterations, Clinicopathological Characteristics, and Gene Expression

We then evaluated the relationship between CNV alterations in 10 m6A regulatory genes and clinicopathological features of HNSCC patients. Clinical staging was divided into I–IV according to the HNSCC guidelines of the American Joint Committee on Cancer (23). The results showed that alterations to m6A regulatory genes were significantly related to age, gender, and clinical stage (*P* < 0.05) (Table 1). In other words, being aged <60 years, male, with high-grade clinical staging were factors more related to CNV events involving 10 m6A regulatory genes.

**Table 1:** Clinical pathological parameters of HNSCC patients with or without CNV of m6A regulatory genes.

**Variable**		**With CNV events**	**Without CNV events**	**Total**	**chi-square value**	***P***
Age	< =60	223	13	236	4.205	**0.0402**
	>60	216	27	243		
Gender	Male	329	22	351	6.462	**0.0110**
	Female	110	18	128		
Clinical Stage	Stage I	14	5	19	9.623	**0.0220**
	Stage II	83	9	92		
	Stage III	93	9	102		
	Stage IV	249	17	266		
Stage M	M0	424	39	463	1.63E-29	1
	M1	5	0	5		
	MX	10	1	11		
Stage N	N0	209	26	235	4.620	0.2018
	N1	74	6	80		
	N2	141	8	149		
	N3	7	0	7		
	NX	8	0	8		
Stage T	T1	28	5	33	4.369	0.2243
	T2	125	15	140		
	T3	118	9	127		
	T4	167	11	178		
	TX	1	0	1		

*With or without CNV events: Cases have CNV or do not have CNV, confirmed by TCGA database. Ambiguous variables (Nx and Mx) were excluded from chi-square test. Bold values indicate P < 0.05 with significantly statistical difference*.

**Figure 4:**
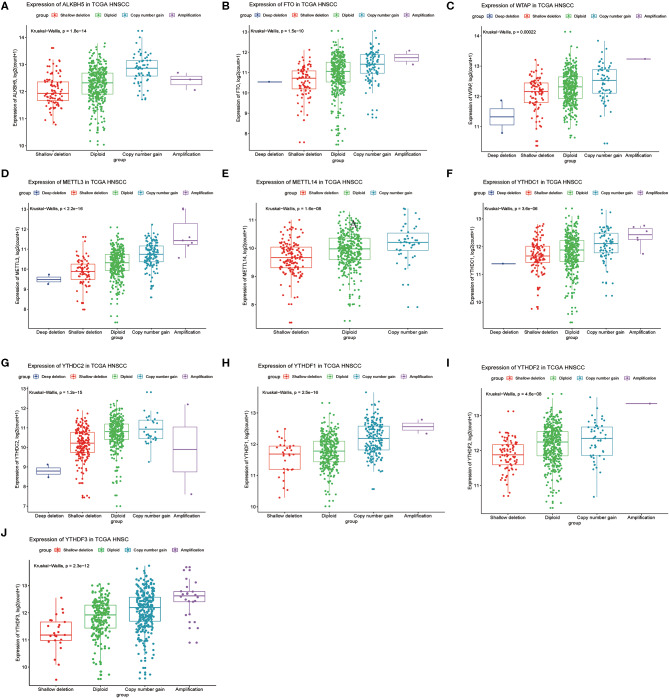
Association between m6A regulatory gene CNV patterns and genetic expression. **(A)** ALKBH5, **(B)** FTO, **(C)** WTAP, **(D)** METTL3, **(E)** METTL14, **(F)** YTHDC1, **(G)** YTHDC2, **(H)** YTHDF1, **(I)** YTHDF2, **(J)** YTHDF3.

Next, we analyzed relationships between CNV patterns and gene expression of the 10 m6A regulatory genes. As can be seen from Figure 4, there was a statistically significant relationship between CNV patterns and gene expression. With CNV events from deletion to amplification, gene expression of the 10 m6A regulatory genes also showed an upward trend; that is, gene expression increased with increased CNV amplification and decreased with CNV deletion.

### Association Between m6A Regulatory Gene CNVs and HNSCC Patient Survival

**Figure 5:**
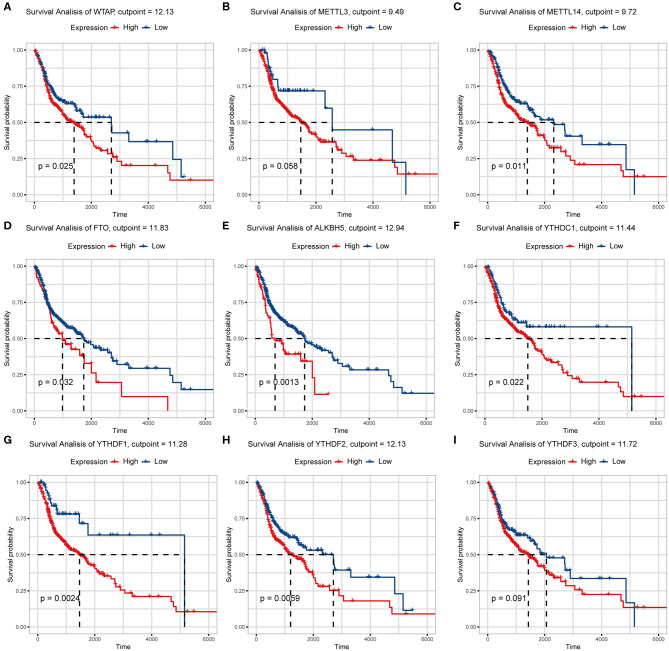
Relationship between expression of nine m6A regulatory genes and overall survival. **(A)** WTAP, **(B)** METTL3, **(C)** METTL14, **(D)** FTO, **(E)** ALKBH5, **(F)** YTHDC1, **(G)** YTHDF1, **(H)** YTHDF2, **(I)** YTHDF3.

**Figure 6:**
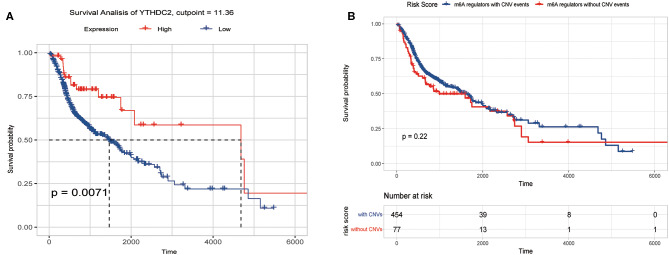
Overall survival of HNSCC patients with or without CNV of m6A regulatory genes and YTHDC2 expression. **(A)** Relation between YTHDC2 expression and overall survival. **(B)** Relation between with or without CNVs and overall survival.

We previously studied relationships between CNV patterns and m6A regulatory gene expression. The more extensive the CNV, the more the gene was expressed. We studied relationships between expression levels of m6A regulatory genes and the survival rate of HNSCC patients and explored the prognostic value of m6A regulatory genes. Our analysis revealed that expression of the 10 m6A regulatory genes was mostly related to the prognosis of HNSCC patients. Patients with high expression of *ALKBH5, FTO, METTL14, WTAP, YTHDC1, YTHDF1*, and *YTHDF2* had lower OS (Figures 5A–H), and patients with higher *YTHDC2* expression showed greater OS (Figure 6A). Furthermore, when HNSCC patients were grouped with or without CNV events for survival analysis, no statistically significant results were obtained (Figure 6B). It could be speculated that the occurrence of CNV events does not directly affect the prognosis of HNSCC patients, but rather that they affect the prognosis of HNSCC patients by influencing the expression of genes. Meanwhile, univariate and multivariate analyses demonstrated that increased expression of *ALKBH5* and *YTHDC2* were independent risk factors for OS in patients with HNSCC (Table 2).

**Table 2:** Univariate and multivariate COX regression analysis of various prognostic parameters for HNSCC cohort.

**Risk factors**	**Univariate**		**Multivariate**	
	**HR(95% CI)**	***P***	**HR(95% CI)**	***P***
Age	1.02(1.01-1.03)	**0.00123**	1.02(1.012-1.0404)	**0.000268**
Gender(male/female)	0.765(0.57-1.03)	0.0747	0.8668(0.6336-1.1858)	0.371157
Clinical M(M0/M1)	4.63(1.71-12.6)	**0.00263**	4.8295(1.744-13.3741)	**0.002444**
Clinical N(N0/N1-N3)	1.24(0.945-1.63)	0.12		
Clinical T(T1-T2/T3-T4)	1.31(0.97-1.76)	**0.0783**	0.7723(0.9535-1.7586)	0.097974
Clinical stage(stage i-ii/stage iii-iv)	1.24(0.889-1.74)	0.203		
METTL3(high/low)	1.617(0.371-1.031)	**0.0655**	1.1450(0.5020-1.5194)	0.631672
WTAP(high/low)	1.478(0.5028-0.9104)	**0.00987**	0.9995(0.6687-1.4971)	0.998031
METTL14(high/low)	1.552(0.4774-0.8693)	**0.00402**	1.3650(0.4864-1.1036)	0.136612
ALKBH5(high/low)	1.726(0.4062-0.8264)	**0.0026**	1.7626(0.3845-0.8371)	**0.004286**
FTO(high/low)	1.437(0.4907-0.9875)	**0.0423**	1.0381(0.6568-1.4128)	0.848160
YTHDF1(high/low)	2.554(0.2183-0.7019)	**0.00164**	1.7128(0.3056-1.1156)	0.103340
YTHDF2(high/low)	1.489(0.5073-0.8896)	**0.00549**	1.0431(0.6480-1.4183)	0.832774
YTHDF3(high/low)	1.251(0.5915-1.08)	0.144		
YTHDC1(high/low)	1.592(0.4346-0.9073)	**0.0132**	1.2575(0.4966-1.2734)	0.340161
YTHDC2(high/low)	0.4903(1.204-3.456)	**0.00806**	0.3896(1.4864-4.4323)	**0.000720**

*Ambiguous variables (Nx, Mx, N/A and discrepancy) were deleted. Bold values indicate P < 0.05 with significantly statistical difference*.

Next, by further analysis, no significant differences were observed between different subgroups based on the CNVs of the 10 m6A regulatory genes (Figure S2).

### Machine Learning and IHC Analysis

**Figure 7:**
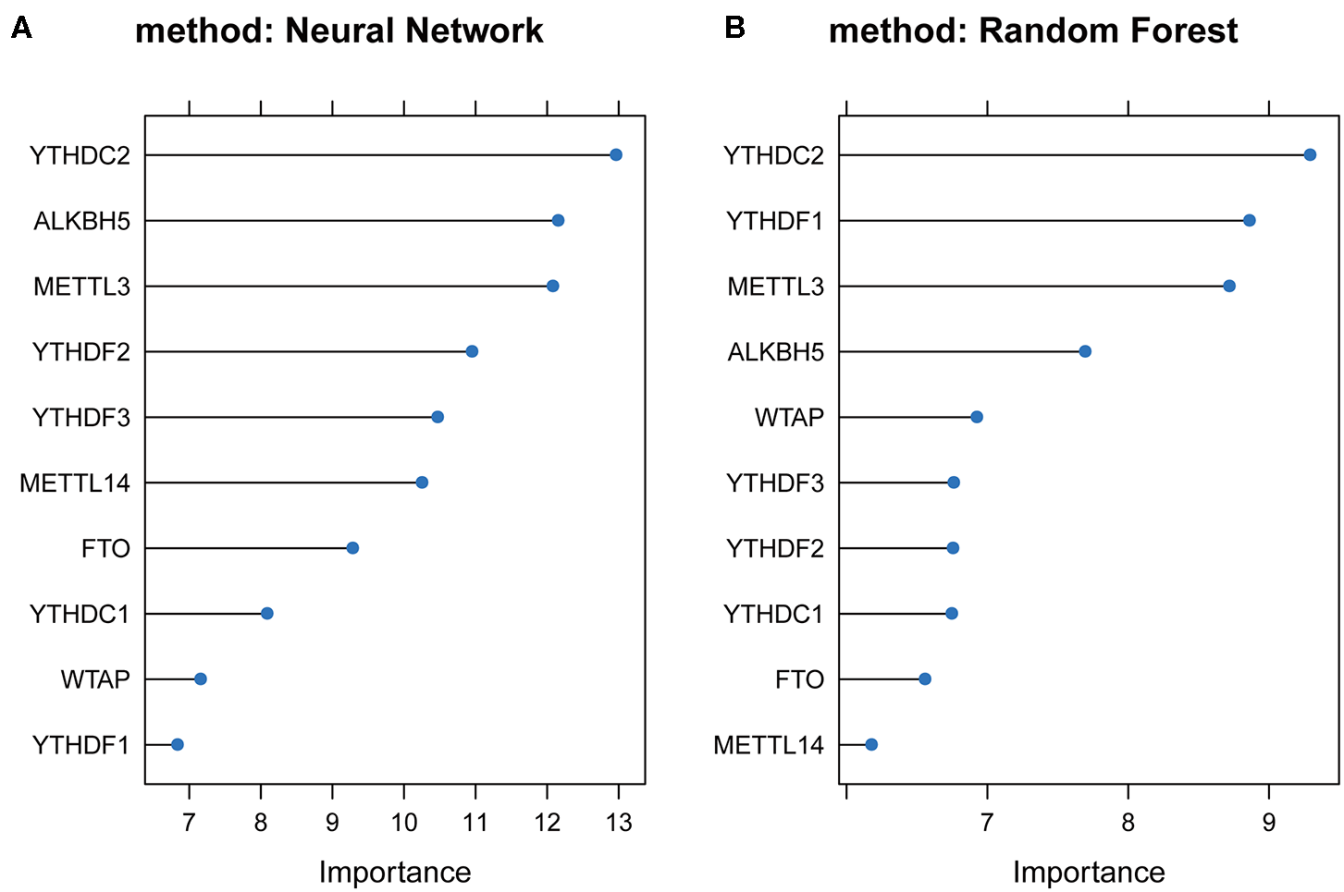
Plots of neural network and random forest for 10 m6A regulatory genes. **(A)** Rank chart for the 10 m6A regulatory genes by Neural network. **(B)** Rank chart of the 10 m6A regulatory genes by Random Forest.

To study the important characteristics of the 10 genes, we used a neural network and random forest to build predictive models. As can be seen from Figure 7, in both models, the *YTHDC2* gene was consistently ranked the highest among the 10 genes. Combined with the results of the previous analysis (Figure 2A), among the 10 genes, the incidence of CNV events involving *YTHDC2* was second only to *YTHDF3*, and *YTHDC2* gene expression and survival prognosis results were also significant. However, association between the expression level of *YTHDF3* and survival prognosis was not statistically significant (Figure 5I). Therefore, *YTHDC2* was selected as the most prognostically important locus of the 10 m6A regulatory genes in HNSCC.

**Figure 8:**
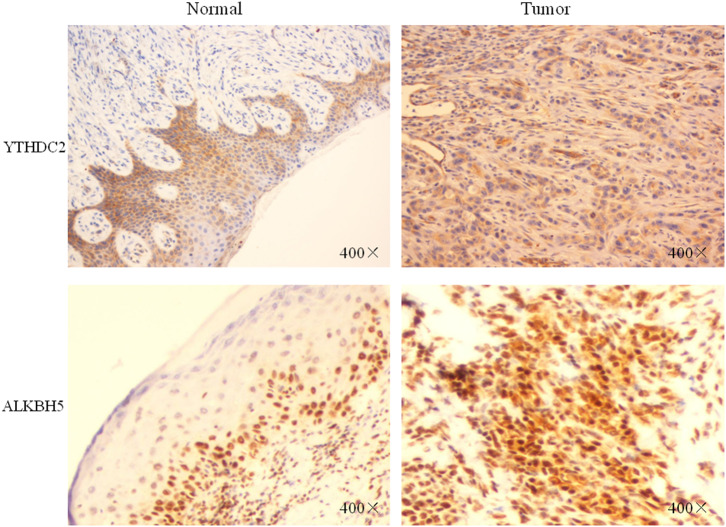
The protein expression of YTHDC2 and ALKBH5 in oral squamous cell carcinoma and normal oral tissues.

Furthermore, we performed IHC staining for *YTHDC2* and *ALKBH5* proteins in 20 pairs of normal and oral squamous cell carcinoma tissues to confirm such findings (Figure 8). These results were consistent with our predictions, indicating that *YTHDC2* and *ALKBH5* were expressed more highly in malignant HNSCC tissues than in normal oral tissues.

### GSEA Analysis

**Figure 9:**
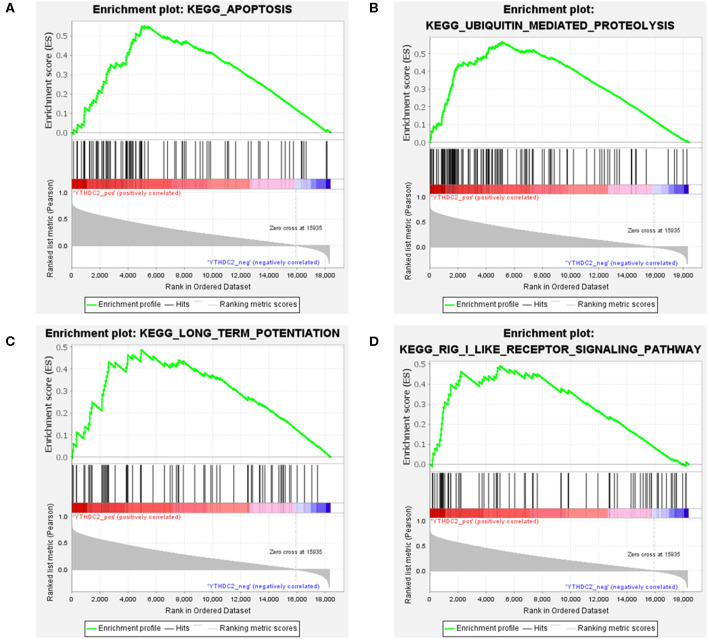
GSEA results of different expression levels of YTHDC2. Gene set enrichment plots of **(A)** apoptosis, **(B)** ubiquitin-mediated proteolysis, **(C)** long-term potentiation, and **(D)** rig-i-like receptor signaling pathways related to low METTL3 mRNA level in the HNSCC samples.

**Figure 10:**
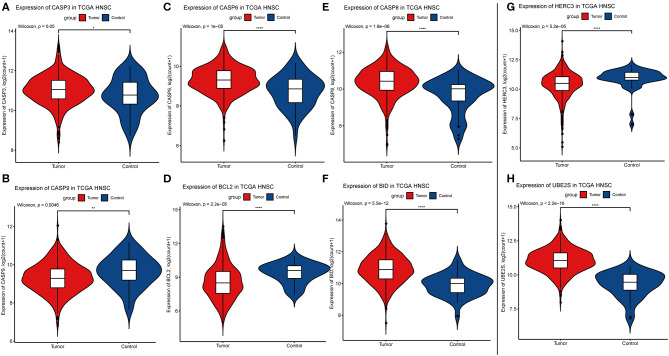
Expression of genes related to apoptosis and ubiquitin-mediated proteolysis. **(A)** CASP3, **(B)** CASP9, **(C)** CASP6, **(D)** BCL2, **(E)** CASP8, **(F)** BID, **(G)** HERC3, **(H)** UBE2S.

Considering the importance of *YTHDC2* in the methylation process, we decided to explore the role of *YTHDC2* dysfunction in the pathogenesis of HNSCC. We examined enriched gene sets in samples with different *YTHDC2* mRNA expression levels. GSEA analysis implied that high expression of *YTHDC2* is associated with certain key pathways, such as apoptosis, ubiquitin-mediated proteolysis, long-term potentiation, and rig-i-like receptor signaling pathways, revealing the underlying mechanisms involved in HNSCC pathogenesis (Figure 9). To validate our findings, we examined the expression of several genes associated with these pathways. We found that apoptosis-related genes *caps3, 6, 8, 9, Bcl-2, Bid, UBE2S*, and *HERC3* associated with ubiquitin-mediated proteolysis were significantly different in HNSCC tumor tissues compared to normal tissues (Figure 10). The GSEA results were partially validated.

## Discussion

m6A is the most abundant chemical modification of mRNA, which is regulated by m6A “writer,” “eraser,” and “reader” proteins (8, 24, 25). As an important complement to the central dogma, the study of m6A-mediated RNA methylation and its role in cancer has only just commenced. To date, little is known regarding the role of m6A in HNSCC. Only a single study has reported that m6A is associated with nasopharyngeal carcinoma in which m6A-mediated ZNF750 repression facilitates NPC progression (26). Due to the complexity of m6A-Seq and m6A-methylated RNA immunoprecipitation technologies, many studies have chosen alterative methods to assess genetic alterations to m6A regulatory genes, indirectly exploring relationships between m6A status and human disease. In this study, we applied bioinformatics methods to analyze genetic alteration in 10 m6A genes, their relationships with the clinical features of HNSCC, and their prognostic value based on a TCGA database.

In our cohort of 506 HNSCC cases, the CNV frequency involving the 10 m6A regulatory genes was similar to that reported in ccRCC (22), but much higher in AML (21), suggesting that m6A dysfunction may play a more important role in the carcinogenesis of HNSCC than that of AML. Furthermore, “reader” genes *YTHDC2* and *YTHDC3* exhibited higher incidences of CNVs than other genes in our HNSCC samples, which is different from that of ccRCC (22). This suggests that “readers” may be more important than “writers” and “erasers” in HNSCC. However, “erasers” *FTO* and *ALKBH5* have been shown to be more important in glioblastoma (27), AML (28), and BRC (29). This implies tissue specificity of the “writers,” “erasers,” and “readers” in different tumors. Unlike in AML (21) and ccRCC (22), the CNV events in the HNSCC samples here led to gains in copy number (1109/980). The most obvious gain and loss genes were “readers,” which further demonstrated the importance of “reader” genes in m6A processing.

Mutations in CNV are usually associated with different clinicopathological features. In the ccRCC cohort, m6A regulatory gene alterations were significantly related to higher Fuhrman nuclear grading. In AML patients, bone marrow blast numbers, white blood cell count, and cytogenetic risk are significantly associated with m6A gene alterations. In our HNSCC samples, the alterations to m6A regulatory genes were significantly correlated with age, sex, and clinical stage. This indicated that alterations in m6A CNV may show different clinical characteristics in different tumors. In the future, it will be necessary to study the characteristics of m6A mutations in different malignancies. Similar to ccRCC samples, in our study, copy number gains were correlated with higher mRNA expression and vice versa.

We evaluated the role of m6A regulatory genes in the survival of patients with HNSCC. In contrast toccRCC samples and BRC samples with only two and three m6A genes associated with OS, here there were eight m6A regulatory genes that were significantly correlated with patient OS. In our study, patients with high expression of *ALKBH5, FTO, METTL14, WTAP, YTHDC1, YTHDF1*, and *YTHDF2* predicted poor patient prognosis. In BRC cohort, high expression levels of *YTHDF1, YTHDF3*, and *KIAA1429* predicted unfavorable patient prognosis. The two populations presented almost completely different prognostic genes. In the AML cohort, patients without m6A mutation had longer survival times. In HNSCC cases, we observed longer OS with diploid or copy number gain, which is different from the situation in AML but similar to that of ccRCC. These results showed that survival of patients with different types of tumors is related to different CNV patterns and different m6A regulatory gene abundance, which is a very attractive feature of m6A epigenetics.

In addition, the expression level of *ALKBH5* is a promising independent prognostic factor for HNSCC, suggesting that *ALKBH5* can be used as a new biomarker of HNSCC. In agreement with our study, some groups have reported that *ALKBH5* could serve as a novel prognostic biomarker that predicts a poor prognosis in colorectal (30) and pancreatic cancers (31).

Finally, we observed another interesting phenomenon. The HNSCC samples with higher *YTHDC2* expression were associated with longer-term survival, which is in contrast to the other m6A genes. *YTHDC2* has been identified as a frequently mutated gene in pancreatic cancer patients and plays a role in tumor metastasis by increasing the translational efficiency of HIF-1α (32, 33). Our IHC staining showed that the expression of *YTHDC2* in tumors was higher than that in normal tissues (Figure 8). Furthermore, by neural network and random forest methods, we found that *YTHDC2* was the most important gene among the 10 m6A regulatory genes. These results suggest that *YTHDC2* might play an important role in HNSCC. However, why did patients with higher *YTHDC2* expression have better prognoses? It has been found that *YTHDC2* is an m6A-binding protein that plays critical roles during spermatogenesis (34). The translation efficiency of *Smc3* (target *YTHDC2* in spermatogenesis) is significantly decreased in *YTHDC2*–/– mice compared to *YTHDC2* +/+ animals. In addition, the testes and epididymis from adult *YTHDC2*–/– mice are smaller in size than wild type (*YTHDC2* +/+). These results show that high *YTHDC2* expression is beneficial for spermatogenesis, which might partly explain why high expression of *YTHDC2* was associated with prolonged OS. However, more work is needed to explore such a mechanism.

The development of HNSCC is related to a series of cancer-related pathways that become uncontrolled. In our HNSCC population, we found that high levels of *YTHDC2* mRNA expression are related to apoptotic activation and the ubiquitin-mediated proteolysis pathway, which are two very important cellular processes in the development of HNSCC. *METTL3* and *METTL14* are the most widely studied m6A regulatory genes in various cancers (24, 35). To date, only one study has reported that m6A is associated with HNSCC, in which *METTL3*-mediated *ZNF750* repression facilitates NPC progression. *METTL3* can promote apoptosis in gastric (36) and breast cancer cells (37) and is associated with ubiquitin-dependent process in pancreatic cancer cells (38). These pathways are similar to those involving *YTHDC2*. Due to the lack of research concerning *YTHDC2*, further studies are needed to verify our findings. However, there is no doubt that our results point out a new direction for the study of m6A in the pathogenesis and progression of HNSCC.

## Conclusion

In HNSCC patients, a majority of highly expressed m6A regulatory genes is associated with poor OS, in particular *ALKBH5*, whereas *YTHDC2* was associated with better prognosis. We identified, for the first time, the expression characteristics and prognosis of m6A regulatory genes in patients with HNSCC. Our results will provide a direction for further exploration of the pathogenesis of HNSCC.

## Data Availability Statement

Publicly available datasets were analyzed in this study. This data can be found here: https://xenabrowser.net/datapages/.

## Ethics Statement

All clinical data, sequence profiles, and CNV data were extracted using UCSC-XENA and the TCGA database, which is open to the public under certain conditions. Therefore, it can be confirmed that all written informed consent forms are available. The study using human oral squamous cell carcinoma tissue and oral mucosa samples for IHC staining was approved by the Ethics Committee of Shandong Provincial Hospital affiliated to Shandong First Medical University. We obtained written informed consent from all participants.

## Author Contributions

XCZ and XYZ mainly performed experiments and data analysis. LD performed IHC of clinical specimens. JH and YL provided intellectual support and expertise in the field of cancer pathology. XCZ, ZC, and SX wrote the manuscript. JH and ZY designated all experiments in this study. All authors contributed to the critical review of the manuscript.

## Conflict of Interest

The authors declare that the research was conducted in the absence of any commercial or financial relationships that could be construed as a potential conflict of interest.

## Supplementary Material

Figure S1 The types of mutations in the 10 m6A regulatory genes associated with HNSCC and the 10 most common genes.

Figure S2 Correlations between survival probability and m6A CNV patterns. **(A)** ALKBH5, **(B)** FTO, **(C)** WTAP, **(D)** METTL3, **(E)** METTL14, **(F)** YTHDC1, **(G)** YTHDC2, **(H)** YTHDF1, **(I)** YTHDF2, **(J)** YTHDF3.

## Abbreviations

m6A: N6-methyladenosine
HNSCC: head and neck squamous cell carcinoma
CNV: copy number variation
TCGA: The Cancer Genome Atlas
GEO: Gene Expression Omnibus
ccRCC: clear cell renal cell carcinoma
AML: acute myeloid leukemia
OS: overall survival
GSEA: gene set enrichment analysis
IHC: immunohistochemistry.

## References

[1] Haddad RI Shin DM. Recent advances in head and neck cancer. The New England journal of medicine. (2008) 359:1143–54. 10.1056/NEJMra0707975 18784104

[2] Bray F Ferlay J Soerjomataram I Siegel RL Torre LA Jemal A Global cancer statistics. Cancer J Clin. (2011) 61:69–90. 10.3322/caac.20107 21296855

[3] Chen W Zheng R Baade PD Zhang S Zeng H Bray F Cancer statistics in China 2015. Cancer J Clin. (2016) 66:115–32. 10.3322/caac.21338 26808342

[4] Siegel RL Miller KD Jemal A Cancer statistics, 2015. CA Cancer J Clin. (2015) 65:5–29. 10.3322/caac.21254 25559415

[5] Hashibe M Brennan P Chuang SC Boccia S Castellsague X Chen C. Interaction between tobacco and alcohol use and the risk of head and neck cancer. Pooled analysis in the International Head and Neck Cancer Epidemiology Consortium. Cancer Epidemiol Biomarkers Prev. (2009) 18:541–50. 10.1158/1055-9965 19190158 PMC3051410

[6] Fu Y Dominissini D Rechavi G He C. Gene expression regulation mediated through reversible m(6)A RNA methylation. Nat Rev Genet. (2014) 15:293–306. 10.1038/nrg3724 24662220

[7] Chen XY Zhang J Zhu JS The role of m(6)A RNA methylation in human cancer. Mol Cancer. (2019) 18:103 10.1186/s12943-019-1033-z 31142332 PMC6540575

[8] Cao G Li HB Yin Z Flavell RA. Recent advances in dynamic m6A RNA modification. Open Biol. (2016) 6:160003. 10.1098/rsob.160003 27249342 PMC4852458

[9] Gallagher EJ LeRoith D. Obesity and diabetes. The increased risk of cancer and cancer-related mortality. Physiol Rev. (2015) 95:727–48. 10.1152/physrev.00030.2014 26084689 PMC4491542

[10] Wei W Ji X Guo X Ji S. Regulatory role of N(6) -methyladenosine (m(6) A) methylation in RNA processing and human diseases. J Cell Biochem. (2017) 118:2534–43. 10.1002/jcb.25967 28256005

[11] Huang Y Su R Sheng Y Dong L Dong Z Xu H. Small-Molecule targeting of oncogenic FTO demethylase in acute myeloid leukemia. Cancer Cell. (2019) 35:677–91.e610. 10.1016/j.ccell.2019.03.006 30991027 PMC6812656

[12] Cui Q Shi H Ye P Li L Qu Q Sun G. m(6)A RNA methylation regulates the self-renewal and tumorigenesis of glioblastoma stem cells. Cell Rep. (2017) 18:2622–34. 10.1016/j.celrep.2017.02.059 28297667 PMC5479356

[13] Liu J Ren D Du Z Wang H Zhang H Jin Y. m(6)A demethylase FTO facilitates tumor progression in lung squamous cell carcinoma by regulating MZF1 expression. Biochem Biophys Res Commun. (2018) 502:456–64. 10.1016/j.bbrc.2018.05.175 29842885

[14] Ma JZ Yang F Zhou CC Liu F Yuan JH Wang F. METTL14 suppresses the metastatic potential of hepatocellular carcinoma by modulating N(6) -methyladenosine-dependent primary MicroRNA processing. Hepatology. (2017) 65:529–43. 10.1002/hep.28885 27774652

[15] Cai X Wang X Cao C Gao Y Zhang S Yang Z. HBXIP-elevated methyltransferase METTL3 promotes the progression of breast cancer via inhibiting tumor suppressor let-7g. Cancer Lett. (2018) 415:11–19. 10.1016/j.canlet.2017.11.018 29174803

[16] Mayakonda A Lin DC Assenov Y Plass C Koeffler HP. Maftools: efficient and comprehensive analysis of somatic variants in cancer. Genome Res. (2018) 28:1747–56. 10.1101/gr.239244.118 30341162 PMC6211645

[17] Mermel CH Schumacher SE Hill B Meyerson ML Beroukhim R Getz G. GISTIC2.0 facilitates sensitive and confident localization of the targets of focal somatic copy-number alteration in human cancers. Genome Biol. (2011) 12:R41. 10.1186/gb-2011-12-4-r41 21527027 PMC3218867

[18] Kassambara A Kosinski M survminer: Drawing Survival Curves using 'ggplot2'. R package version 0.4.3. (2018). Available online at: https://CRAN.R-project.org/package=survminer.

[19] A Package for Survival Analysis in S_. version 2.38. (2015). Available online at: https://CRAN.R-project.org/package=survival.

[20] Warde-Farley D Donaldson SL Comes O Zuberi K Badrawi R Chao P. The GeneMANIA prediction server. biological network integration for gene prioritization and predicting gene function. Nucleic acids Res. (2010) 38(Web Server issue):W214–220. 10.1093/nar/gkq537 20576703 PMC2896186

[21] Kwok CT Marshall AD Rasko JE Wong JJ Genetic alterations of m(6)A regulators predict poorer survival in acute myeloid leukemia. J Hematol Oncol. (2017) 10:39 10.1186/s13045-017-0410-6 28153030 PMC5290707

[22] Zhou J Wang J Hong B Ma K Xie H Li L. Gene signatures and prognostic values of m6A regulators in clear cell renal cell carcinoma - a retrospective study using TCGA database. Aging. (2019) 11:1633–47. 10.18632/aging.101856 30877265 PMC6461179

[23] Sridharan S Thompson LDR Purgina B Sturgis CD Shah AA Burkey B Early squamous cell carcinoma of the oral tongue with histologically benign lymph nodes. A model predicting local control and vetting of the eighth edition of the American Joint Committee on Cancer pathologic T stage. Cancer. (2019) 125:3198–207. 10.1002/cncr.32199 31174238 PMC7723468

[24] Liu J Yue Y Han D Wang X Fu Y Zhang L. A METTL3-METTL14 complex mediates mammalian nuclear RNA N6-adenosine methylation. Nat Chem Biol. (2014) 10:93–5. 10.1038/nchembio.1432 24316715 PMC3911877

[25] Ping XL Sun BF Wang L Xiao W Yang X Wang WJ. Mammalian WTAP is a regulatory subunit of the RNA N6-methyladenosine methyltransferase. Cell Res. (2014) 24:177–89. 10.1038/cr.2014.3 24407421 PMC3915904

[26] Zhang P He Q Lei Y Li Y Wen X Hong M. m(6)A-mediated ZNF750 repression facilitates nasopharyngeal carcinoma progression. Cell Death Dis. (2018) 9:1169. 10.1038/s41419-018-1224-3 30518868 PMC6281568

[27] Zhang S Zhao BS Zhou A Lin K Zheng S Lu Z. m(6)A demethylase ALKBH5 maintains tumorigenicity of glioblastoma stem-like cells by sustaining FOXM1 expression and cell proliferation program. Cancer Cell. (2017) 31:591–606 e596. 10.1016/j.ccell.2017.02.013 28344040 PMC5427719

[28] Li Z Weng H Su R Weng X Zuo Z Li C. FTO plays an oncogenic role in acute myeloid leukemia as a N(6)-methyladenosine RNA demethylase. Cancer Cell. (2017) 31:127–41. 10.1016/j.ccell.2016.11.017 28017614 PMC5234852

[29] Zhang C Zhi WI Lu H Samanta D Chen I Gabrielson E. Hypoxia-inducible factors regulate pluripotency factor expression by ZNF217- and ALKBH5-mediated modulation of RNA methylation in breast cancer cells. Oncotarget. (2016) 7:64527–42. 10.18632/oncotarget.11743 27590511 PMC5323097

[30] Liu X Liu L Dong Z Li J Yu Y Chen X. Expression patterns and prognostic value of m(6)A-related genes in colorectal cancer. Am J Trans Res. (2019) 11:3972–91. 31396313 PMC6684930

[31] Cho SH Ha M Cho YH Ryu JH Yang K Lee KH. ALKBH5 gene is a novel biomarker that predicts the prognosis of pancreatic cancer: A retrospective multicohort study. Ann Hepato—Pancr Surg. (2018) 22:305–9. 10.14701/ahbps.2018.22.4.305 30588520 PMC6295372

[32] Fanale D Iovanna JL Calvo EL Berthezene P Belleau P Dagorn JC. Germline copy number variation in the YTHDC2 gene: does it have a role in finding a novel potential molecular target involved in pancreatic adenocarcinoma susceptibility? Exp Opin Thera Targ. (2014) 18:841–50. 10.1517/14728222.2014.920324 24834797

[33] Tanabe A Tanikawa K Tsunetomi M Takai K Ikeda H Konno J. RNA helicase YTHDC2 promotes cancer metastasis via the enhancement of the efficiency by which HIF-1alpha mRNA is translated. Cancer Lett. (2016) 376:34–42. 10.1016/j.canlet.2016.02.022 26996300

[34] Hsu PJ Zhu Y Ma H Guo Y Shi X Liu Y. Ythdc2 is an N(6)-methyladenosine binding protein that regulates mammalian spermatogenesis. Cell Res. (2017) 27:1115–27. 10.1038/cr.2017.99 28809393 PMC5587856

[35] Bokar JA Shambaugh ME Polayes D Matera AG Rottman FM. Purification and cDNA cloning of the AdoMet-binding subunit of the human mRNA (N6-adenosine)-methyltransferase. RNA. (1997) 3:1233–47. 9409616 PMC1369564

[36] He H Wu W Sun Z Chai L. MiR-4429 prevented gastric cancer progression through targeting METTL3 to inhibit m(6)A-caused stabilization of SEC62. Biochem Biophys Res Commun. (2019) 517:581–7. 10.1016/j.bbrc.2019.07.058 31395342

[37] Wang H Xu B Shi J. N6-methyladenosine METTL3 promotes the breast cancer progression via targeting Bcl-2. Gene. (2020) 722:144076. 10.1016/j.gene.2019.144076 31454538

[38] Taketo K Konno M Asai A Koseki J Toratani M Satoh T. The epitranscriptome m6A writer METTL3 promotes chemo- and radioresistance in pancreatic cancer cells. Int J Oncol. (2018) 52:621–9. 10.3892/ijo.2017.4219 29345285

