# Supplementary figures and images for: Analysis of Genetic Alteration Signatures and Prognostic Values of m6A Regulatory Genes in Head and Neck Squamous Cell Carcinoma
Source: Front Oncol. 2020 May 29;10:718. doi: 10.3389/fonc.2020.00718 (PMC7273190; doi:10.3389/fonc.2020.00718)

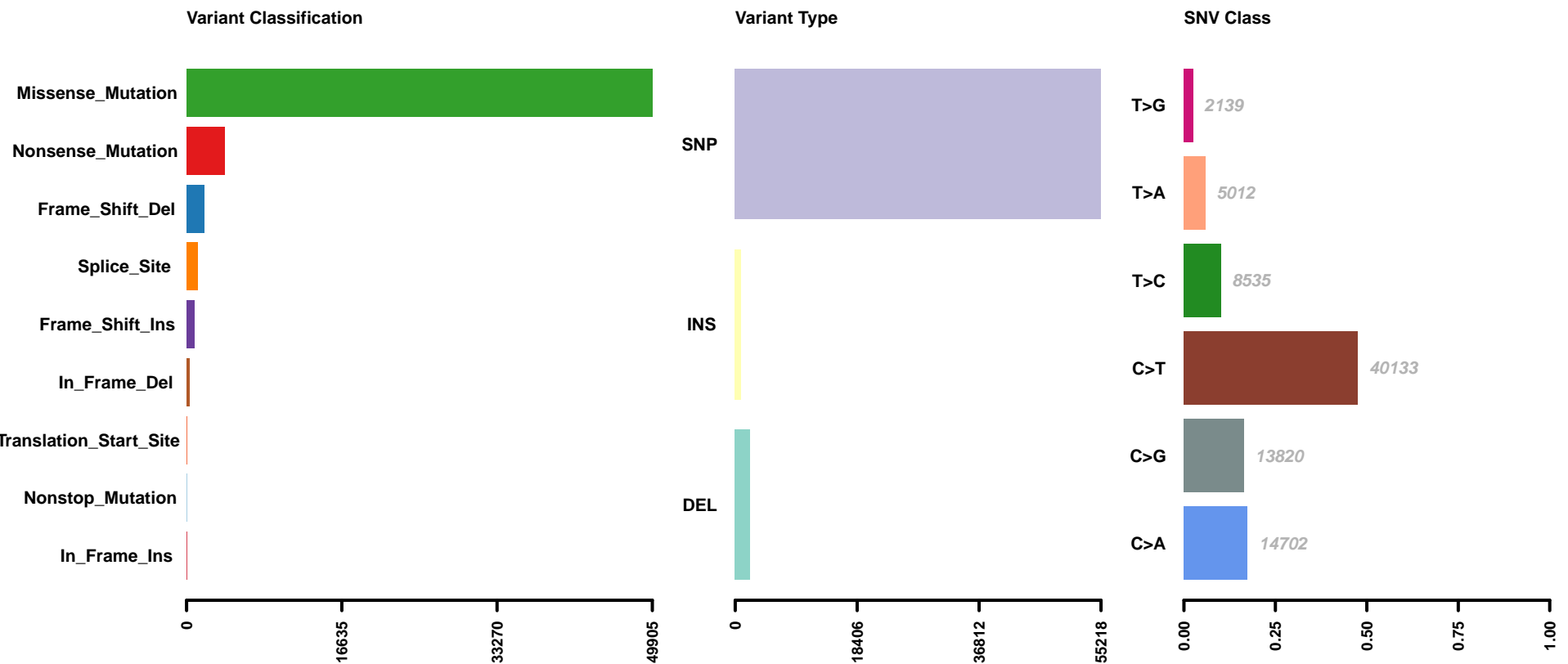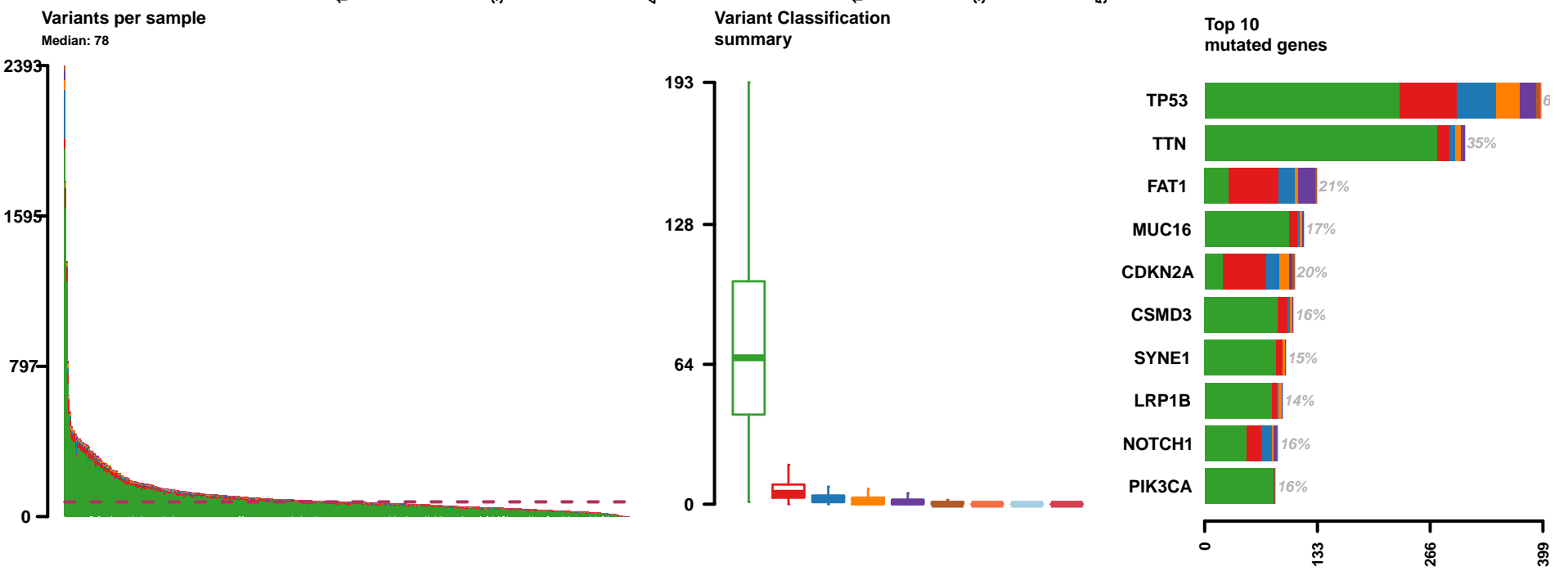

Supplement: Figure S1 — The types of mutations in the 10 m6A regulatory genes associated with HNSCC and the 10 most common genes. [file Image_1.pdf]

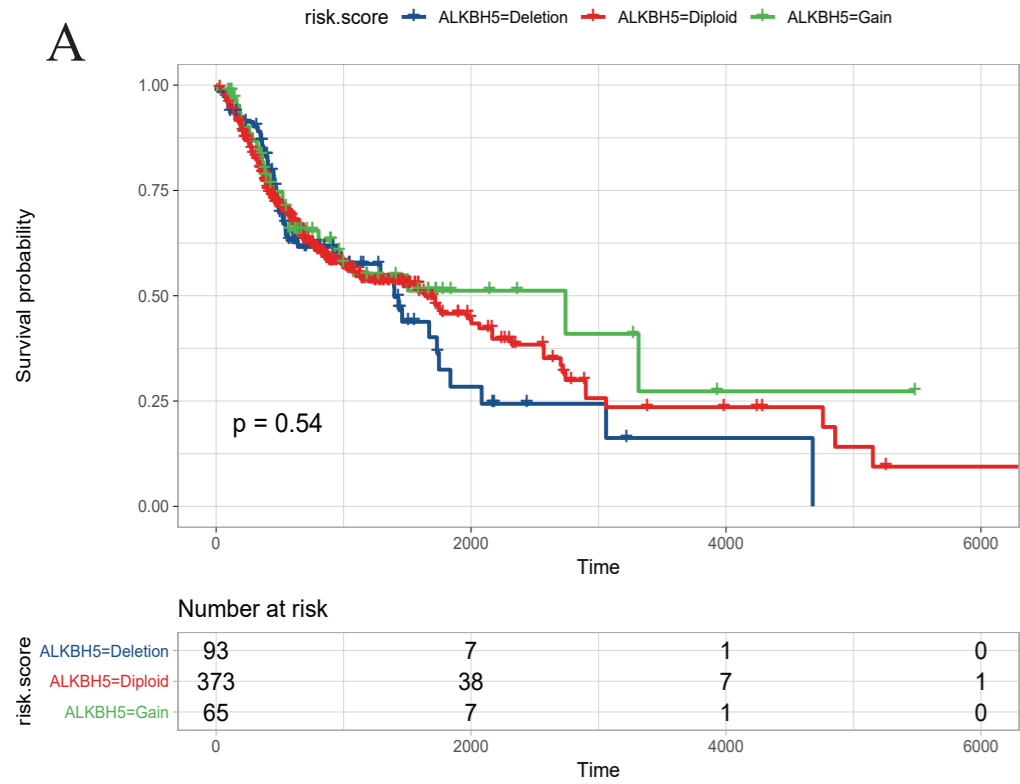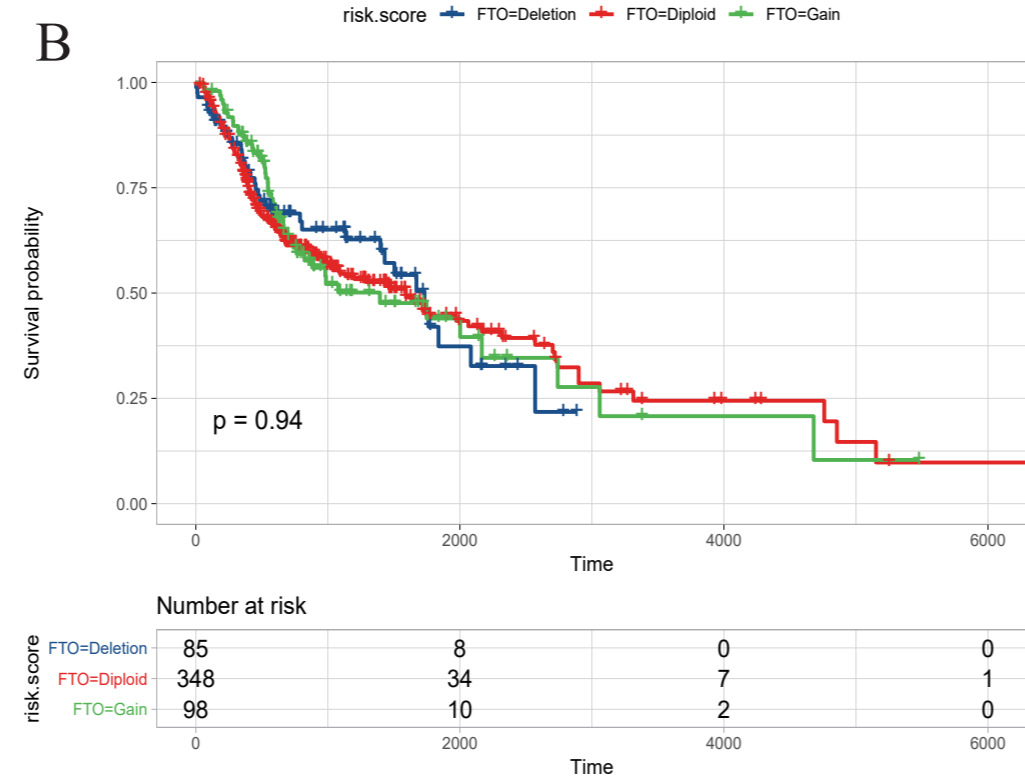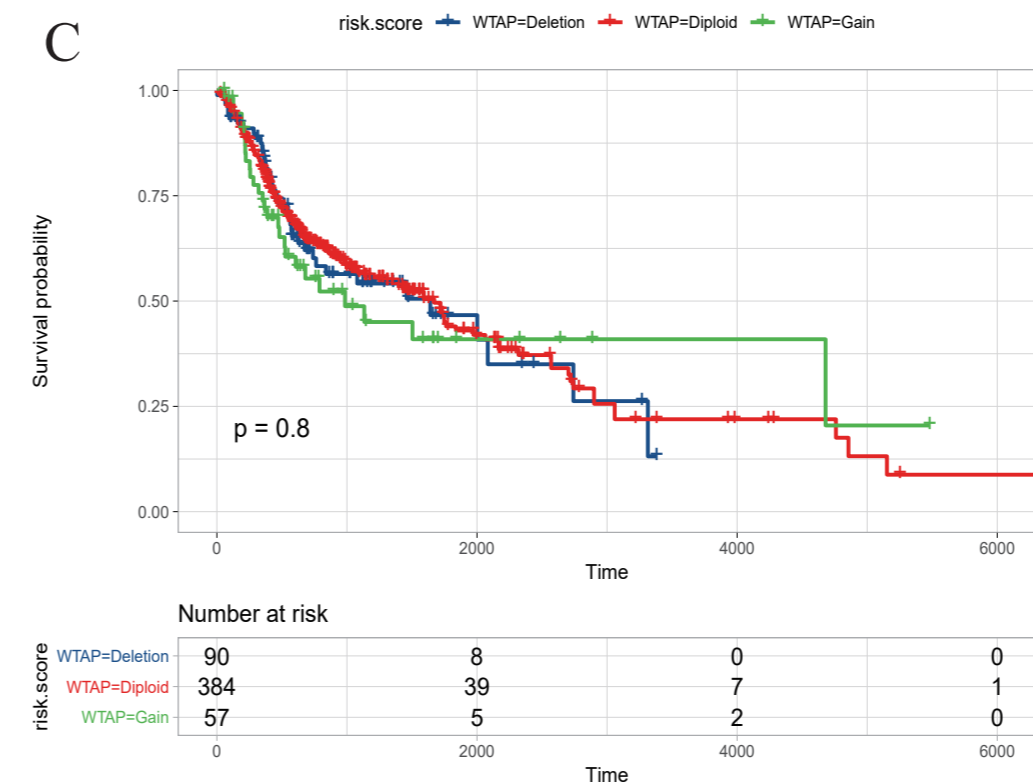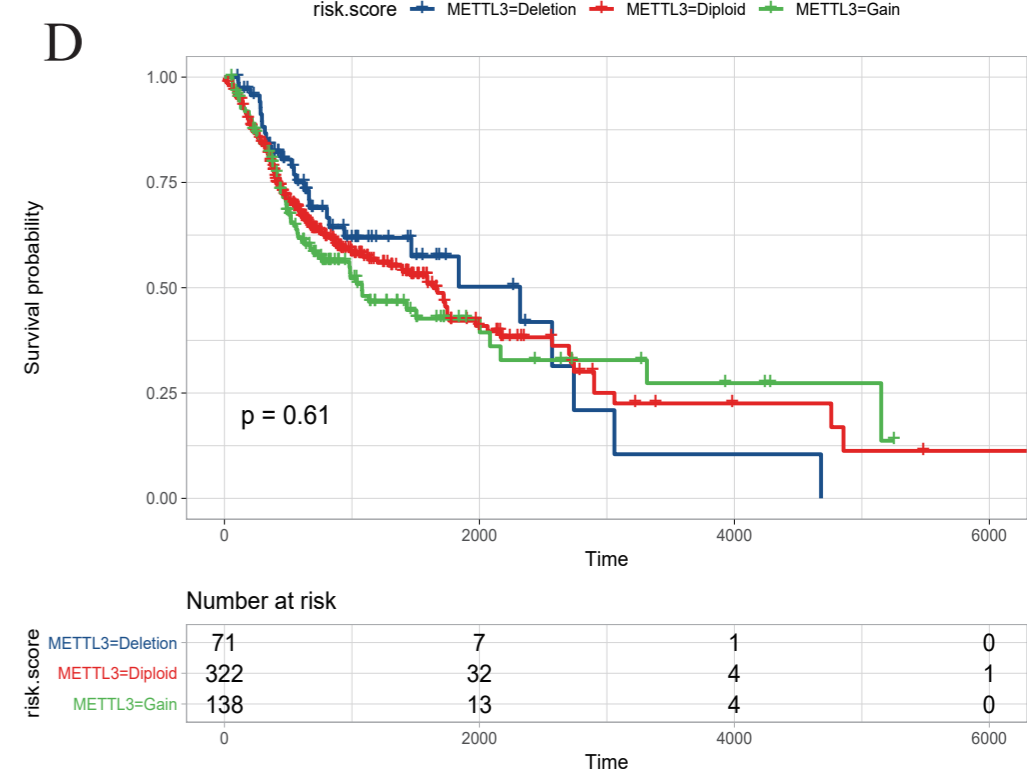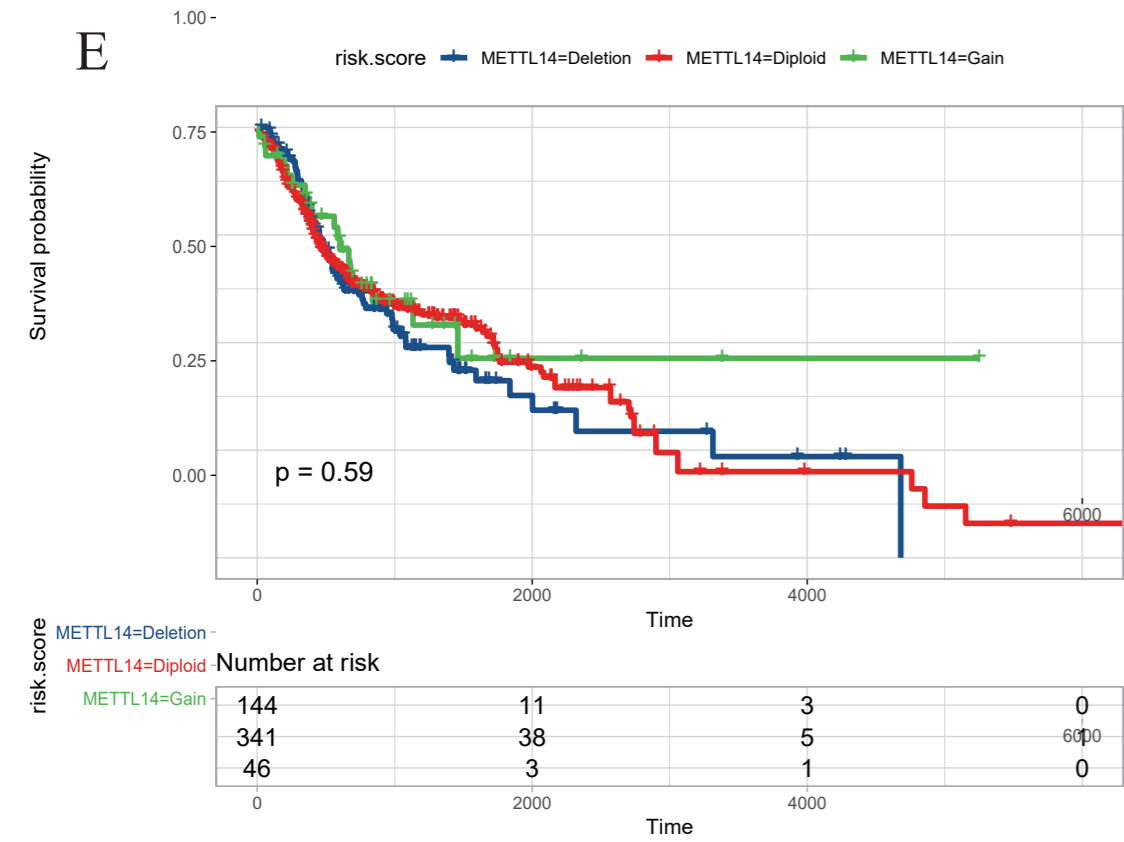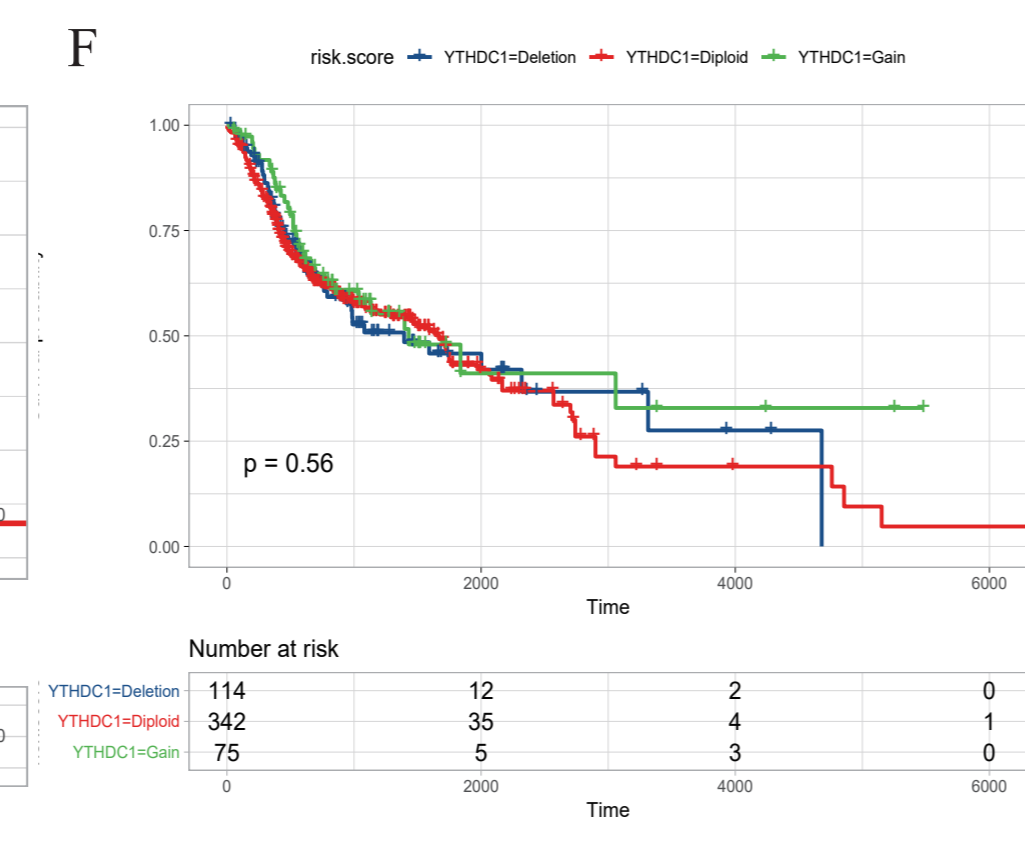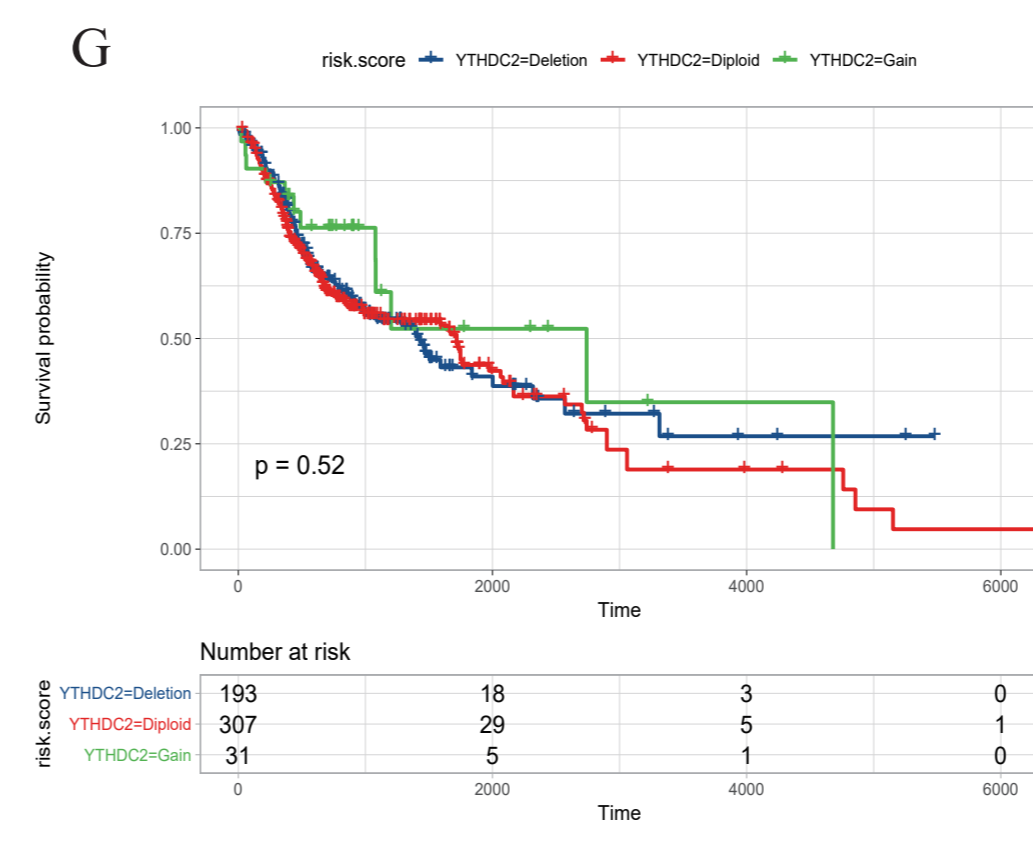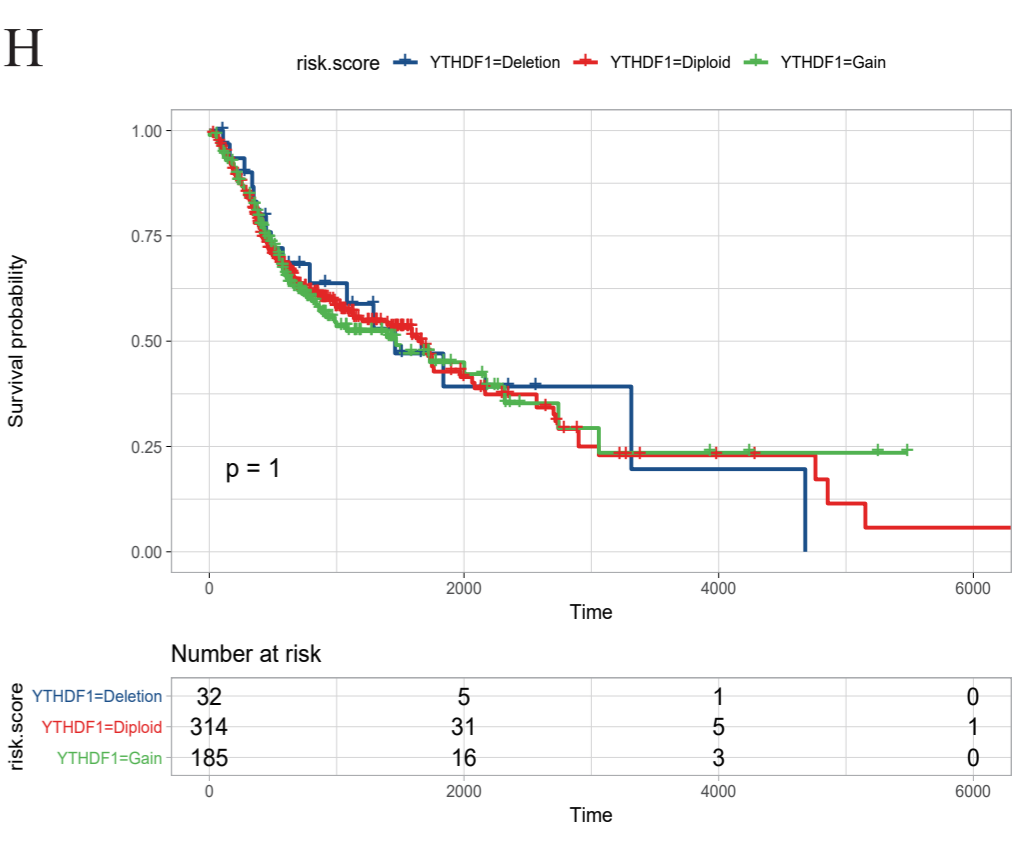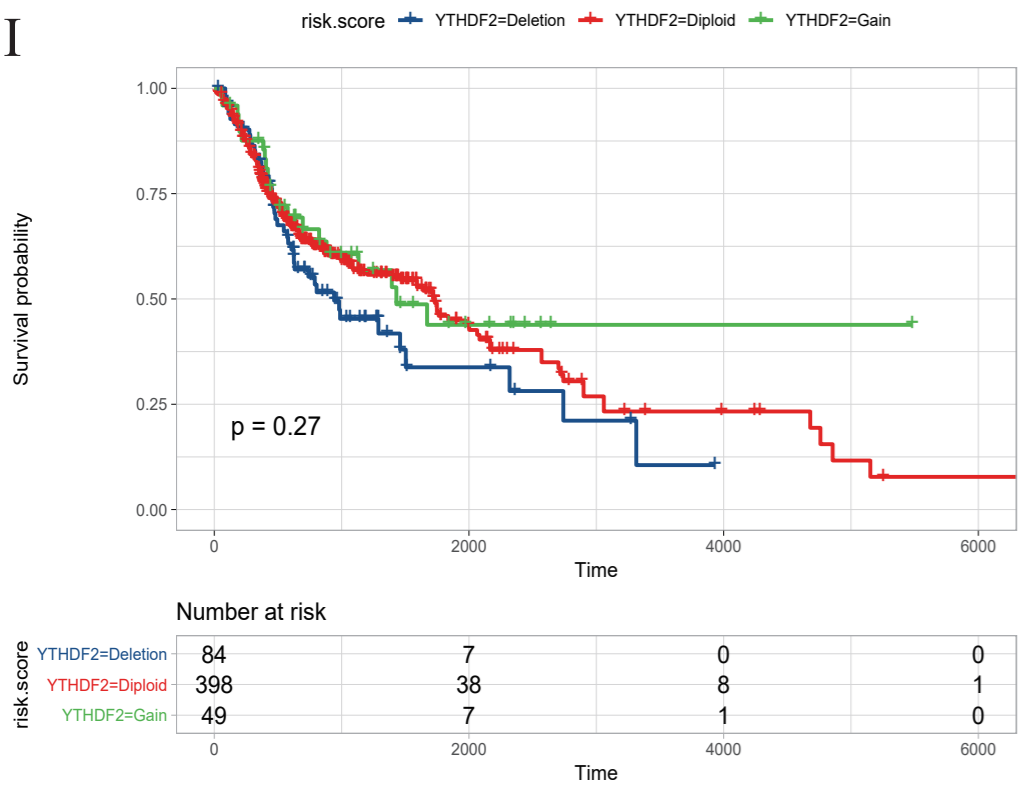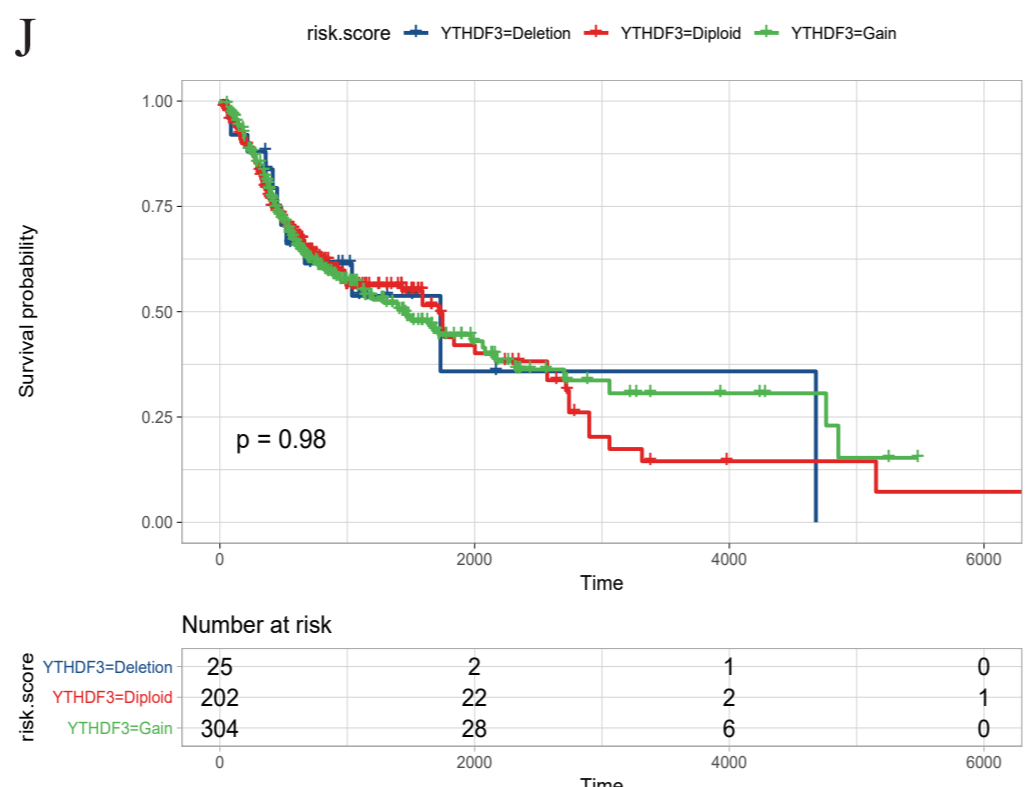

Supplement: Figure S2 — Correlations between survival probability and m6A CNV patterns. (A) ALKBH5, (B) FTO, (C) WTAP, (D) METTL3, (E) METTL14, (F) YTHDC1, (G) YTHDC2, (H) YTHDF1, (I) YTHDF2, (J) YTHDF3. [file Image_2.pdf]
